# Thymoma may explain the confusion: a case report

**DOI:** 10.1186/s13256-021-03178-6

**Published:** 2021-12-16

**Authors:** Abdelkhaleq Maaroufi, Naoufal Assoufi, Mohamed Amine Essaoudi, Jamal Fatihi

**Affiliations:** 1 Department of Internal Medicine B, Mohamed V Military Hospital, Faculty of Medical Sciences, University Mohammed V, Rabat, Morocco; 2 Department of pathology, Mohamed V Military Hospital, Faculty of Medical Sciences, University Mohammed V, Rabat, Morocco

**Keywords:** Inflammatory myopathy, Myasthenia gravis, Association, Thymoma

## Abstract

**Background:**

The association of inflammatory myopathy and myasthenia gravis is a rarely described entity whose clinical presentation has always been intriguing because of the great clinical similarity between these two pathologies. The presence of a thymic pathology often explains this combination, whose mechanisms are very complex.

**Case presentation:**

A 56-year-old woman of North African origin, was hospitalized to explore the Raynaud phenomenon associated with proximal muscle weakness, pain, and arthralgia. There was no rash, and neuromuscular examination had revealed proximal tetraparesis and mild neck weakness. Tendon reflexes were normal. There was no abnormal nail fold capillaroscopy. A significant titer of muscle enzymes had been shown on blood tests, and autoimmune screening for myositis-specific and myositis-associated autoantibodies was negative. Electromyography had shown a myopathic pattern, and muscle biopsy confirmed an inflammatory myopathy. Although steroids were introduced, the clinical course was unsatisfactory; ophthalmic and bulbar symptomatology appeared. The association of myasthenia gravis was confirmed by an elevated level of serum acetylcholine receptor. A chest computed tomography scan had identified a thymoma. Treated with prednisone, pyridostigmine, and thymectomy, the patient’s clinical and biological evolution was favorable.

**Conclusion:**

This case illustrates an exceptional association of two entities and the difficulty encountered during their diagnosis and treatment. The management of these two diseases is different, so it is essential to recognize this concomitant presentation.

## Introduction

Inflammatory myopathy (IM) and myasthenia gravis (MG) are well-known and distinct neuromuscular diseases. Myasthenia gravis is an autoimmune disorder of neuromuscular transmission involving the production of autoantibodies directed against the nicotinic acetylcholine receptor [1]. These antibodies disrupt neuromuscular transmission and cause muscle weakness [2].

Inflammatory myopathies are a heterogeneous group of muscle disorders of autoimmune origin, characterized by muscle weakness, elevated creatine kinase (CK), and myopathic pattern on electromyography (EMG). Usually, a muscle biopsy confirms the diagnosis. In some patients, myositis’ clinical and histological features fulfill criteria of polymyositis or dermatomyositis [3].

Clinical, electrophysiological, and biological features distinguish these two neuromuscular pathologies. There have been many reports of patients with both MG and IM. The coexistence of MG and IM might be associated with thymoma [4]. The first case was described in 1942; since then, more than 40 cases have been reported with the combination of MG, IM, and thymoma [5, 6].

This paper describes a patient with MG and IM associated with thymoma and comments on the relevant features that helped diagnose and manage the patient.

## Case presentation

A 56-year-old woman of North African origin, with no medical and surgical history, was admitted to the Department of Internal Medicine for investigation of a Raynaud phenomenon evolving for 3 months, associated with inflammatory arthralgias involving the wrists, elbows, and ankles; the patient also reported pain and progressive proximal muscle weakness in her lower limbs when climbing stairs, which extended to the upper limbs. There was no rash. Neuromuscular examination revealed proximal tetraparesis, mild neck flexion, and extension weakness. Strength at the neck was 4/5; at the shoulder 3/5; at the wrist 4/5; at the hips 2/5; and at the ankle 4/5; dorsiflexion/plantar flexion was normal. Tendon reflexes were normal. A few days after her hospitalization, the patient developed slight dysphonia and dysphagia; then this symptomatology was accentuated over time.

**Fig. 1:**
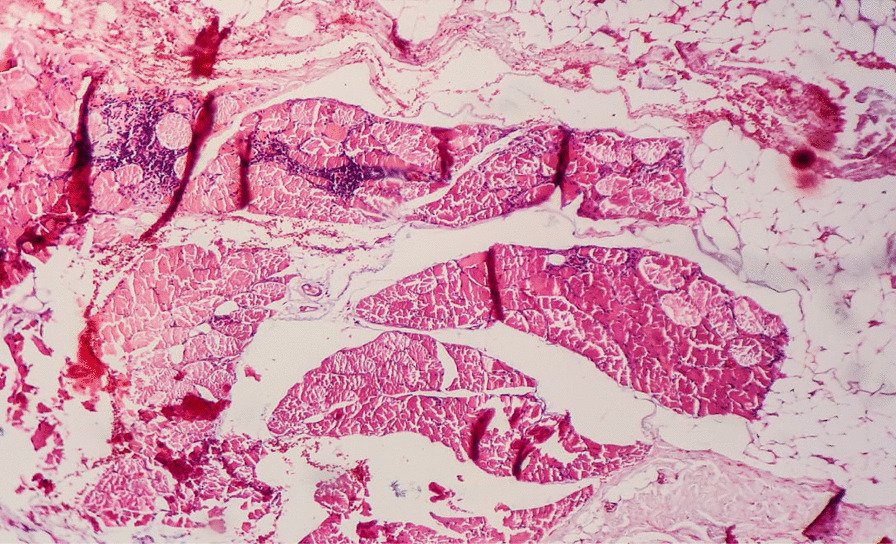
Skeletal muscle showing degenerative changes with chronic inflammatory infiltrate; hematoxylin and eosin (HE) ×10

Nail fold capillary microscopy was normal. Blood tests showed significant levels of muscle enzymes [creatinine kinase (CK) 40,000 IU/L]. Autoimmune screening for myositis-specific and myositis-associated autoantibodies was negative. Electromyography (EMG) showed a decrement in compound muscle action potential on repetitive stimulation and clear signs of myopathy. The diagnosis of inflammatory myopathy was confirmed by muscle biopsy, which showed myositis (Fig. 1). The patient was treated with prednisone 60 mg daily; we observed a slight improvement in muscle weakness and a significant decrease in CK serum level.

**Fig. 2:**
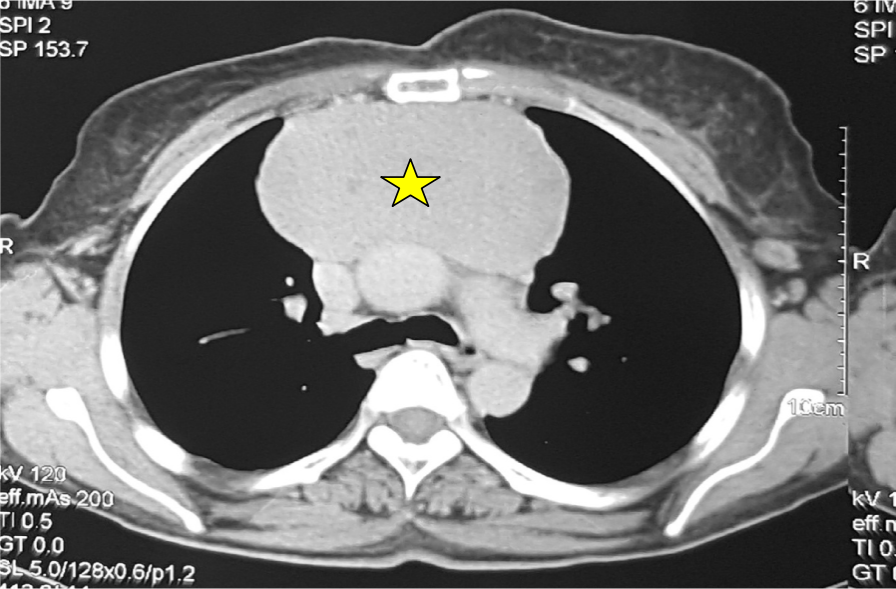
Chest CT showing an anterosuperior mediastinal mass (the yellow star represents the tumor mass)

**Fig. 3:**
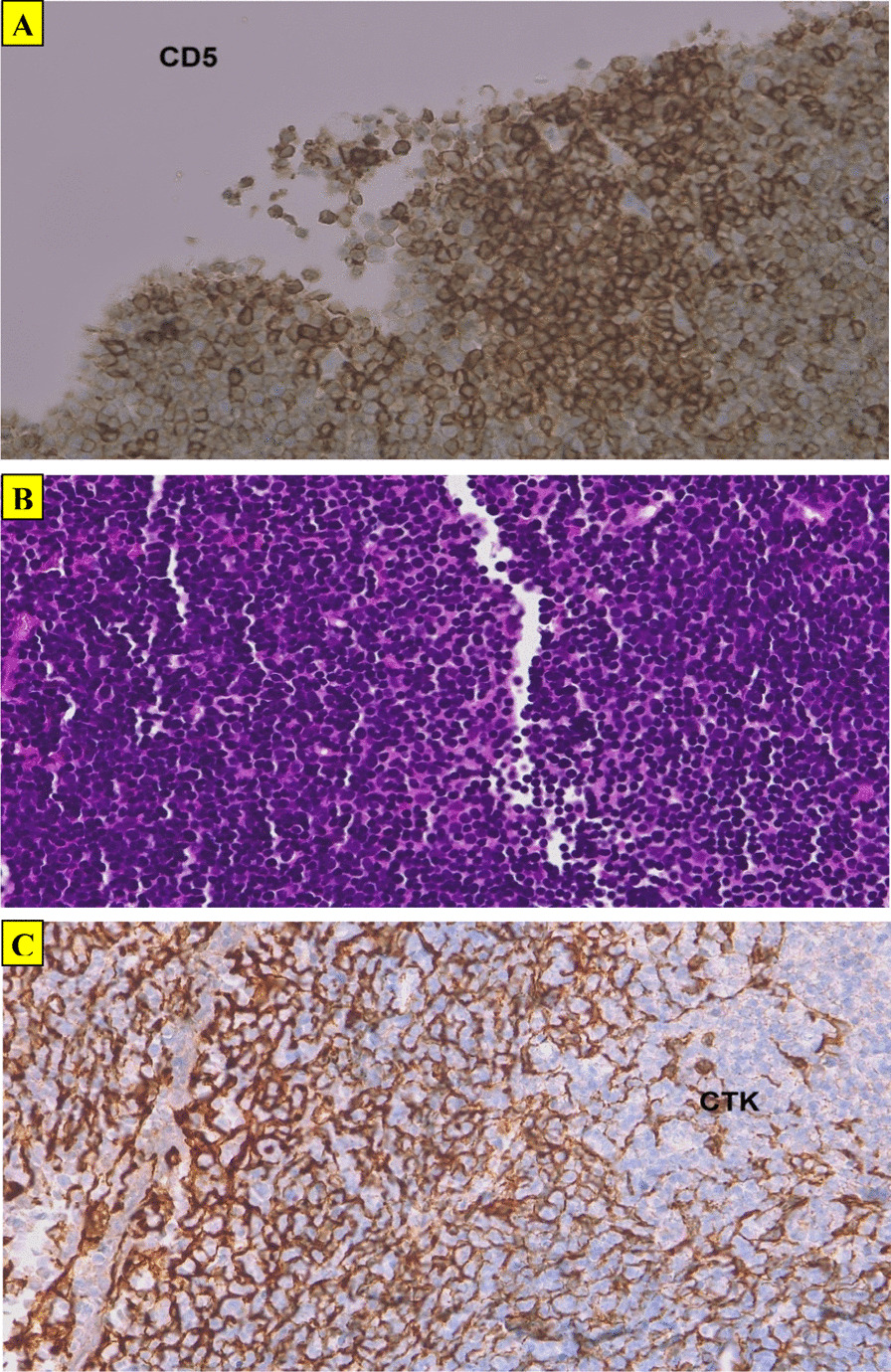
**A**CD5 antibody staining the lymphocytes diffusely (CD5 ×25). **B** Epithelial cells surrounded by prominent lymphoid stroma (HE ×25). **C** Pancytokeratin (×25) highlighting epithelial cells

A month later, she developed intermittent diplopia, ptosis, and mild dysarthria during the conversation. Serum acetylcholine receptor (AChR) antibodies were positive (5.59 nmol/L), and muscle-specific kinase antibodies were negative. A broader workup performed with computed tomography (CT) of the chest revealed a large anterior mediastinal mass (114 × 57 × 100 mm) (Fig. 2), which a subsequent biopsy revealed as B1-type thymoma (Fig. 3).

Inflammatory myositis associated with myasthenia gravis was diagnosed. Pyridostigmine in combination with steroids was started, with significant improvements in generalized weakness, ptosis, and dysarthria. The patient underwent thymectomy as part of the additional treatment. Currently, she is on a low dose of steroids and pyridostigmine and is asymptomatic. The features of myositis have not relapsed.

## Discussion

We have described the case of a patient with a rare association of two entities: MG and IM, whose autoimmune and inflammatory pathogenesis is common; both occur with a high frequency in association with other autoimmune diseases [7, 8]. Some clinical features should attract the clinician’s attention to the presence of another concomitant neuromuscular disorder, namely MG, superimposed by myositis or vice versa.

Initially, our patient presented with muscular symptomatology, muscle weakness, and myalgia, which favored inflammatory myopathy confirmed by the elevated level of creatine kinases, myopathic changes on EMG, and muscle biopsy results; thereby, IM was diagnosed. However, the clinical evolution of the patient under adequate treatment was not satisfying, especially the occurrence of other deficits, such as diplopia, ptosis, ophthalmoparesis, and fatigability, which are not typical symptoms in inflammatory myopathies. Even ocular symptoms are considered exclusion criteria [9]. This prompted us to demand a serum AChR antibody assay, which was positive with a high titer. The diagnosis was concluded to be myositis with concomitant MG. A chest CT completed the investigation, revealing a histological type B1 thymoma.

The coexistence of MG and IM may be associated with thymoma as a paraneoplastic phenomenon. It is generally rare, and only a few cases have been reported in case reports published in the English literature [10]. The association of IM and MG in the absence of thymoma is less common [11]. Myasthenia gravis usually precedes IM in published case reports, but both can be concomitant; rarely, IM may be the first manifestation of the neuromuscular disorder [2], as reported in our case. Thymic pathology is known to be associated with MG; thymic hyperplasia is reported in nearly 80% of MG patients with thymectomy [12]. It has been further noted that myasthenic symptoms can be resolved by thymectomy, especially in patients with thymoma. [12].

Some types of autoimmune myositis, specifically cases of dermatomyositis, are associated with cancers, including thymic tumors [13]. Myositis can involve cardiac muscle, resulting in heart failure and arrhythmias, either exclusively or skeletal muscle [14]. Thymic involvement may explain this association; in patients with thymoma, 40% have one or more paraneoplastic autoimmune conditions, about 20–25% of which is myasthenia gravis [15]. Thus, mediastinum imaging either by CT or magnetic resonance imaging (MRI) is essential in assessing a patient with myositis MG [12].

In our case, this reflection allowed us to readjust our diagnosis, thereby rectifying our treatment by adding pyridostigmine and thymectomy with a dramatic improvement of the patient and her life quality.

## Conclusion

Our case aims to make clinicians aware of the importance of recognizing this rare association of IM and MG, its various patterns of involvement in the muscles and neuromuscular junction, and its diagnostic and vital therapeutic implications in improving prognosis. This association should give rise to the thymoma hypothesis and a specific follow-up program to rule out malignancy.

## Data Availability

The datasets supporting the conclusions of this article are included within the article and its additional files.

## Abbreviations

IM: Inflammatory myopathy
MG: Myasthenia gravis
CK: Creatine kinase
EMG: Electromyography
AChR: Acetylcholine receptor
HE: Hematoxylin and eosin
